# Numeracy, adult education, and vulnerable adults: a critical view of a neglected field

**DOI:** 10.1007/s11858-020-01155-9

**Published:** 2020-04-06

**Authors:** Iddo Gal, Anke Grotlüschen, Dave Tout, Gabriele Kaiser

**Affiliations:** 1 University of Haifa, Haifa, Israel; 2 University of Hamburg, Hamburg, Germany; 3 Australian Council for Educational Research, Adelaide, Australia; 4 Australian Catholic University, Banyo, Australia

**Keywords:** Adult numeracy, Adult education, Mathematics education, Vulnerability, Critical thinking, Literacy skills

## Abstract

This survey paper examines selected issues related to the intersection of three broad scholarly areas: *numeracy*, *adult education*, and *vulnerability*. Numeracy encompasses the ways in which people cope with the mathematical, quantitative, and statistical demands of adult life, and is viewed as an important outcome of schooling and as a foundational skill for all adults. The focus on vulnerability stems from the realization that concerns of policy makers and educators alike often center on populations seen as vulnerable. The paper is organized in five sections. After a brief introduction, Section 2 examines adult numeracy, focusing on five numeracy domains (health, financial, digital, civic, and workplace numeracy), literacy–numeracy linkages, functional and critical aspects of numeracy, and the centrality of numeracy practices, and notes sources of vulnerability for each of these. Section 3 sketches formal, non-formal and informal contexts in which adults learn or develop their numeracy, and examines factors that may be potential sources of vulnerability, including systemic factors and dispositional and affect factors. Section 4 reflects more broadly on the concept of vulnerability, introduces selected aspects of the papers published in this issue of *ZDM Mathematics Education*, and points to findings regarding adult learners who may be deemed vulnerable. The closing section summarizes conclusions and research directions regarding the intersection of the three core domains. Overall, the paper points to emerging research needs and educational challenges that are relevant to scholars, practitioners, and policy makers interested in developing the numeracy of adults as well as in the mathematics education of younger learners.

## Introduction

Adult numeracy is a vital field of interest to societies and economies worldwide, and to the billions of individuals who live in them, and who come from all walks of life and age groups, from young adults and school leavers, all the way to elderly persons. Yet, the area of adult numeracy is under-researched and faces many challenges. At the same time, this area holds much promise from educational, research, and policy perspectives. This survey paper, together with the 16 papers in this special issue of *ZDM Mathematics Education,* examines selected topics at the intersection of three broad scholarly areas: *numeracy*, *adult education*, and *vulnerability*, doing so from a lifelong perspective.

While the term numeracy is used in some countries to describe desired knowledge and skills expected of all school graduates and adults, other expressions, such as quantitative literacy and mathematical literacy are used internationally. The diversity of terms used for numeracy is also complicated by the lack of an equivalent term in some languages. What is meant by numeracy can also vary when applied to school children as opposed to adults.

Adult numeracy is a construct related to the ways people cope with the many mathematical, quantitative, and statistical demands of adult life. Some definitions of *numeracy* emphasize basic computational skills or focus on emergent numeracy at a young age. In this paper, however, numeracy is used broadly to encompass a set of diverse skills, knowledge-bases, dispositions and affect, communication abilities, and practices and behaviors, that range from simple to very advanced, relate to mathematics and statistics, and that individuals need or use in order to engage and manage diverse life situations and tasks in the adult world.

The need to focus on vulnerability in connection with numeracy stems from the realization that many concerns by policy makers and the general public, and many educational efforts related to numeracy or mathematics-related knowledge of adults and young people, focus on *vulnerable* populations (UNESCO 2018) with diverse characteristics. For instance, subgroups in the population may be identified as having low or insufficient numeracy skill levels, which imply a need for further learning or skill upgrading and require relevant or customized educational interventions. Indeed, as reviewed later, large-scale studies show that sizeable proportions of adults and young adults in many countries (including many high-income or developed ones) demonstrate low numeracy (or mathematical literacy).

Although this paper focuses on issues pertaining to adults as learners or users of numeracy, we believe that many of these issues are also pertinent to the learning and teaching of mathematics at the high-school or 'young adult' levels. After all, in many countries, young people leave schools early on, and enter the adult world or the labor market well before age 18. Many countries are expressing interest in mathematical literacy, which is closely linked with adult numeracy. Hence, some of the insights developed in this paper, and the findings and ideas presented in the other papers in this issue of *ZDM Mathematics Education*, will also be of value to educators and researchers interested in mathematics education at the school level, in both STEM (science, technology, engineering, mathematics) and vocational education, and in working with young people and adults deemed vulnerable in such contexts.

No examination of the intersection of the areas of numeracy, adult education, and vulnerability has been published to date. Since each of these three areas is in itself a broad domain with extensive literature, a systematic review of them all is beyond the scope of a single paper. Hence, we focus here on selected sources from multiple disciplines that enable us to sketch each of the three areas in broad strokes, but mainly help to examine their *intersection* and explore sources of potential vulnerability. Furthermore, since issues pertaining to the skills, dispositions, and practices of teachers, programs, and learners are influenced by many factors, the paper combines an educationalist lens with a policy or systemic lens. (Note: terms such as skills, competencies, and abilities are used interchangeably in this paper, given the diversity in their usage in the literature; OECD 2019a; Rychen and Salganic 2003).

With the above in mind, the remainder of this paper is organized in four sections: Sect. 2 examines adult numeracy, and Sect. 3 sketches the terrain of formal, non-formal, and informal contexts in which adults learn or develop their numeracy, but both sections focus on factors that may serve as potential sources of vulnerability. Section 4 reflects more broadly on the concept of vulnerability, introduces selected aspects of the papers published in this issue of *ZDM Mathematics Education*, and points to some findings regarding adult learners who may be deemed vulnerable. Section 5 summarizes conclusions regarding the intersection of the three core areas, and points to emerging needs and future research and development directions.

## About numeracy of adults and young people

This section reviews selected issues, including skill gaps in numeracy, the changing roles of numeracy, links between literacy and language and numeracy, and findings and ideas regarding numeracy practices. This section also discusses an evolving conceptualization of numeracy as both functional and critical, which educators and researchers could take on board in the twenty-first century, and reflects on the intersection of all of the above with the notion of vulnerability (which is expanded on in Sect. 4).

### Numeracy needs and gaps

Increased attention to adult numeracy is manifested in many ways around the globe. In particular, the United Nations Sustainable Development Goals (SDGs) Target 4.6 calls on *all* world countries to "ensure that all youth and a substantial proportion of adults, both men and women, achieve literacy and numeracy" by 2030 (see: https://sustainabledevelopment.un.org/sdg4). The explicit inclusion of numeracy in this goal implies that there are serious gaps regarding numeracy (and literacy) levels across the globe.

The growing acknowledgement and interest in adult numeracy is also evident in the coverage of numeracy and related constructs in large-scale assessment programs, at both the international and national levels. The Adult Literacy and Lifeskills (ALL) survey in the mid-2000s was the first study to explicitly address numeracy, which was later included as a major domain in OECD's Program for the International Assessment of Adult Competencies (PIAAC), which was undertaken between 2008 and 2019 in 39 countries and regions, including seven non-OECD participants. These adult assessments surveyed people aged 16–64. The Program for International Student Assessment (PISA), which focuses on school students aged 15 years old, which was implemented in its latest cycle in 2018 in 79 countries, focused on a similar construct, mathematical literacy (Tout and Gal 2015). These surveys and the many policy-related studies that use their results, highlight the need to address both the numeracy and the literacy skills of a country’s population.

Findings regarding proficiency levels of adults in numeracy (PIAAC) or of young adults in mathematical literacy (PISA), suggest that large numbers of adults and young people, sometimes upwards of 30–60% in middle income countries and 10–40% in high-income countries, have low or very low numeracy skills (OECD 2019a). Furthermore, the overall PIAAC performance in numeracy showed that 7.5% of the adult population were at or below level 1 (the lowest levels) across 39 countries, compared with only 3.7% at the same level in reading literacy (OECD 2019a).

Numeracy has also been included in stand-alone national adult skills surveys developed in some high-income countries, such as the French Information et Vie Quotidienne (IVQ) survey or the UK's Skills for Life survey. England and France tested both literacy and numeracy in two rounds of these surveys, and both reported a *decrease* in numeracy, while literacy skills increased (Jonas 2018).

Going beyond the PIAAC findings, which cover mainly high-income OECD countries, relatively little is known about the distribution of adult numeracy skills in lower-income countries, which are the majority of world countries, given the lack of large-scale comparative data. A notable exception are the results from UNESCO's Literacy Assessment and Monitoring Programme (LAMP), which in 2009–2011 showed that substantial groups of adults in four middle-income countries or national entities (Jordan, Mongolia, Palestine, Paraguay) have very low numeracy skills.

Regarding the preparation of younger populations to tackle numeracy tasks in adulthood, results from the PISA for Development (PISA-D) assessment project (Ward 2018) show an even more alarming pattern. Only about 12% of students across seven middle- or low-income countries (Cambodia, Ecuador, Guatemala, Honduras, Paraguay, Senegal, and Zambia) achieve the *minimum* PISA level of proficiency in mathematical literacy (Level 2), compared with the OECD average of 77%; about 23% achieved the minimum level of proficiency in reading, compared with the OECD average of 80%.

It can thus be assumed that large skill gaps and resulting learning needs regarding numeracy and mathematical literacy exist in most middle- and low-income countries, given the quality of education systems and school dropout rates in such countries (Hanushek and Woessmann 2007). Due to such needs and gaps, many of the papers in this issue of *ZDM Mathematics Education* focus on sub-populations with relatively low numeracy (and literacy) proficiency, since such populations often have to cope with multiple vulnerabilities (Grotlüschen et al. 2016). This section continues to reflect on the purposes served by numeracy, and on numeracy practices and conceptualizations that have implications for a discussion of vulnerability.

### The changing roles and purposes of numeracy in the twenty-first century

The literature describes a range of contexts or situations where foundational skills such as literacy and numeracy are called upon or invoked. The concept of literacy has a long tradition of being understood in plural as *literacies* and the literature refers to literacies as being situated, purposeful, and occurring within specific *literacy domains* (Barton and Hamilton 2000). Parallel developments have occurred in terms of attention to multiple *numeracies*. An early scheme developed by Steen (1990) outlined five purposes for numeracy: *Practical*, (focused on mathematical and statistical knowledge and skills that can help to cope with tasks in daily life); *Professional* (mathematical and statistical skills needed in specific jobs); *Civic* (data and uses that can benefit society); *Recreational* (related to the role of mathematical ideas and processes in games, sports, lotteries, and diverse leisure activities); and *Cultural* (appreciation of mathematical aspects of human culture, such as in artistic artifacts).

Many other projects have looked at the roles and purposes of numeracy over the last few decades. Gal (1997) listed nine contexts that build and elaborate on Steen's scheme: home, recreation, active parenting, personal finance, informed citizenship, social action, workplace, passing tests, and further education. In Australia, the concept of different numeracies was developed from the notion of different literacies mentioned above, and was used in curriculum frameworks for both adults and young people. It centered around four domains (Kindler 1996): Numeracy for practical purposes, Numeracy for interpreting society, Numeracy for personal organization (e.g., money, time, and travel/location), and Numeracy for knowledge, which addressed more formal mathematical skills needed for further study.

Such and related classifications of the range of areas and situations in which adults or young people operate, were condensed into shorter lists in designing the conceptual and assessment frameworks for ALL and PIAAC, as well as PISA. PIAAC's first cycle referred to four contexts of numeracy, everyday life, work, societal, and further learning. The latest PISA cycle referred to personal, occupational, societal, and scientific contexts. Tout and Gal (2015) compared these sets of descriptors and argued that they are highly consistent with each other, with differences due only to relevance to the age of the two target groups.

Research on numeracies in different life domains is continuing to evolve. Interviews with diverse stakeholders in 16 German federal states (Duncker-Euringer 2016) helped identify four specific literacy domains (digital, financial, health, and political) and related uses and practices of literacy in the context of work, family and everyday life, and continuing education. These informed the literacy definitions of the German literacy decade (2016–2026) and the content of national literacy surveys. Based on this work, Grotlüschen et al. (2019a) suggested establishing a system of four numeracy domains: financial numeracy, health numeracy, digital numeracy, and political or civic numeracy.

Indeed, over the last 2 decades, separate conceptual frameworks and findings have developed that support each of the above four domains. These are briefly presented below but are described in more detail in Sect. 4.3.1, where their connection to vulnerability is explained:

- *Financial numeracy* includes many actions related to the critical evaluation of financial information, data, and risk (Lusardi and Mitchell 2011), as opposed to simply reading texts about financial issues. Hence, this term seems more relevant than the well-established domain of financial literacy.
- *Health numeracy* is emerging as an independent field in medical and health sciences research, in parallel to continued interest in health literacy (Golbeck et al. 2005).
- *Digital numeracy* is becoming increasingly important alongside digital literacy, in times of increasing datafication (Steen 1999), public exposure to big data, and the use of algorithms (Michael and Lupton 2016), and alongside growing opportunities to access open data sets and participate in ‘citizen science’ initiatives.
- *Civic numeracy* is emerging as a complex domain that merges mathematics, statistics, and other areas, and involves critical numeracy (Geiger et al. 2015), statistical literacy (Gal 2002), and civic statistics (ProCivicStat Partners 2018).

*Workplace numeracy*, or workplace mathematics, has emerged as a separate area of adult life and has been the focus of much research. This domain must be addressed explicitly, as an additional fifth numeracy domain, since numeracy (and literacy) practices differ in work contexts compared with other life domains. A review of the many publications on workplace numeracy is beyond the scope of the present paper. Risking oversimplification, however, we can summarize that numeracy tasks that people undertake in many types of workplaces, involve much more than basic arithmetic skills or procedural competence with school-based mathematics. For example, based on an employers' survey, Madison and Steen (2003) found that:

Work-related mathematics is rich in data, interspersed with conjecture, dependent on technology, and tied to useful applications. Work contexts often require multi-step solutions to open-ended problems, a high degree of accuracy, and proper regard for required tolerances. None of these features are found in typical classroom exercises (p. 55).

Many sources argue that the impact of changing technology and ICT systems, tools and processes, causes twenty-first century workplaces to require higher STEM skills (Maass et al. 2019) or techno-mathematical literacies (Hoyles et al. 2010), more sophisticated mathematical problem solving skills, the ability to address tasks related to risk and statistics, and critical interpretation of data in various forms, among other trends (FitzSimons 2019). Other research shows, however, that mathematics is becoming increasingly invisible, and sometimes disappears within technological systems (Marr and Hagston 2007; Straesser 2015).

Beyond the five broad numeracy domains or areas listed above, we are witnessing many changes that affect numeracy demands across the lifespan (Griffin and Care 2015; Michael and Lupton 2016). These are brought upon by the rapid rise in the use and daily impact of algorithms, big data, machine learning, artificial intelligence, predictive systems, the availability of open data, and more. Citizens and workers from all walks of life are affected by the changes in both the explicit and transparent mathematical and statistical processes within multiple numeracy domains. Such trends have important implications for educators, as they may both *increase* and *decrease* motivations to invest in further learning of numeracy and mathematics by adults, and should impact policy or curricular decisions regarding the educational content that needs to be taught.

### Connections between numeracy and mathematics and literacy and language

Overall, literacy–numeracy linkages and interdependencies point to possible sources of vulnerability in adult numeracy education. Findings from both PIAAC (OECD 2019a) and PISA (OECD 2019b) show a strong association between performance in reading/literacy and mathematics/numeracy. Hence, this section discusses some of these links and connections.

Solving a numeracy problem situated in the real world often involves a process of reading, interpreting, solving, and communicating in a mathematical manner. As part of such process, it is necessary to understand and use a range of both text-embedded informal and formal linguistic components (both oral and written), as well as mathematical terms, language, symbols, and representations. Literacy aspects are critical in reporting and communicating results. An added complexity, is that the language of mathematics itself is particularly critical as part of the problem-solving process.

There is a broad literature on language and literacy in the mathematics classroom that revolves around classroom discourse and related aspects of the interactions between learners, teachers, textbooks, and learning tasks (Gal 1999; Morgan et al. 2014; Tout 1991). Ellerton (1989), for example, argued that:

Teachers have often assumed that incorrect solutions to problems have arisen from a lack of understanding of mathematical concepts or a deficiency in computational skills. In fact, the errors have been caused by an inadequate understanding of the language of mathematics (p. 95).

However, many scholars have pointed to the need to consider literacy and language (written and spoken) when discussing numeracy as it is practiced in the real world (e.g., in the context of the five domains listed earlier), and pointed to two-way conceptual and pragmatic links between numeracy and mathematics on the one hand, and literacy and language on the other (Baker and Street 1994). Stated informally, there is no literacy without numeracy, and no numeracy without literacy. In this issue of *ZDM Mathematics Education*, several papers argue that language matters for numeracy skills (Prince and Frith 2020) as well as for numeracy practices (Civil et al. 2020; Fonseca 2020; Jorgensen 2020).

An important aspect of the literacy–numeracy linkage was acknowledged early on by the Kirsch and Mosenthal’s construction of literacy as comprised of Prose, Document, and Quantitative dimensions (Kirsch et al. 1998). For example, in many life contexts, quantitative or spatial information is communicated via text or is embedded in text and various types of documents (e.g., timetables, price lists, newspaper articles reporting results of surveys). Practices in life domains listed earlier may involve following instructions about medicine dosages, checking financial information online, or reading newspaper articles with textual descriptions and graphs about ‘burning’ civic issues (ProCivicStat Partners 2018) such as migration trends, global warming, or the spread of the recent Corona-virus pandemic.

Likewise, in the workplace, workers need to read documentation (e.g., about occupational health and safety or operating procedures) that involves data, measurements, or proportions that are integrated into textual materials. Croce and McCormick (2019) argue that cumulative findings in the subfield of ‘disciplinary literacy’ show that professionals do not separate between language and mathematics in their discourse, and there are communities of mathematical discourse within specific functional contexts (e.g., different types of workplaces).

It follows that all key numeracy domains require a wide range of numeracy skills and practices, but are also *predicated on text comprehension and literacy skills* (Gal 1999). General linguistic and text comprehension skills, as well as familiarity with discourse patterns within specific functional and social contexts, can impact the ability of both learners and adults to comprehend, reason about, and communicate regarding real-world numeracy tasks that involve any kind of text, as well as on their in-class work and interactions.

For adult numeracy learners, this complex set of linkages and interdependencies between numeracy and mathematics and literacy and language, points to a range of different sources of vulnerability that need to be understood and explicitly addressed in adult numeracy education. This also has consequences for the diagnosis of the stumbling blocks encountered by each individual adult numeracy learner, since they may have to do with mathematics, or literacy, or language, or their different combinations.

### Numeracy as a (social) practice

The notion of *practices*, when discussing literacy or numeracy, is multifaceted, because the concept is treated in different ways in the literature, referring both to behaviors in a social context, as well as to how, or how often, or how frequent a certain skill is used (i.e., practiced) in diverse contexts. Both notions are briefly reviewed below.

A scholarly discussion on practices began early on, initially in connection with literacy practices (Baker and Street 1994) and evolved under the umbrella of the *new literacy studies* (Barton and Hamilton 2000). The practices perspective assumes that literacy and numeracy are not neutral cognitive skills or knowledge, but are embedded or played out in the real world within a context. The context is characterized by societal factors such as social and power relations, cultural and discourse norms and goals, and opportunities within one's environment or one's ways of engagement with such tasks, i.e., practices (see Reder's Practice-Engagement Theory (1994) or Reder 2020, in this issue).

It has been argued that literacy and numeracy skills and behaviors are taught in formal schooling as if they were neutral (Barton and Hamilton 2000), but a historically informed view shows that dominant literacy practices of the middle and upper classes push aside or devalue the more vernacular practices of lower classes (Yasukawa et al. 2018). Work on numeracy practices evolved from Lave's early research on cognition in practice (Lave 1988), and continued with discussions on (young) adults’ numeracy and conceptualizations of mathematical knowledge and behavior as social practices (e.g., Evans et al. 2017). Such work suggested that skills and how they are acted out in the world may be shaped by a community of practice and by one's levels and ways of engagement with diverse tasks, and as such, should be measured (Coben and Alkema 2017).

Yasukawa et al. (2018) recently summarized the theoretical development of the overarching concept of *numeracy as social practice*, arguing that numeracy is not free of value or context, but embedded in power relations. They base these ideas not only on Street's work, but also on three generations of the Cultural-Historical Activity Theory (CHAT) developed by Vygotsky, Leontiev and Engeström, and on Lave's early work on situated learning.

In contrast, the notion of practices has also been examined, without its societal or cultural aspects, in comparative surveys of adults' skills. Surveys such as ALL and PIAAC include in their background questionnaire a set of frequency-of-use questions about both literacy and numeracy practices in two life contexts, *work practices* and *everyday practices*. For numeracy, questions typically inquire, "how often do you normally…" followed by a list of activities, such as read invoices, bank statements or similar; read plans, maps or graphs; calculate prices, costs or budgets; calculate using fractions, decimals or percentages; read diagrams, charts or tables; use simple formulas or similar; or use higher level mathematics or statistics. While seemingly simplistic, such questions provide important new information about the ways in which numeracy is used in everyday life and at work, and about the correlates of frequency of practices and their association with actual skill levels.

Numerous studies have analyzed PIAAC data based on such questions. For example, Jonas (2018) found that the level of proficiency in numeracy skills and the frequency of engagement in numeracy practices supported one another, i.e., proficient adults perform numerical tasks frequently in their private and working lives, and adults who regularly engage in numeracy practices are more likely to maintain or improve their performance in numeracy. Likewise, PIAAC data showed that employed people engage in mathematical activities less in their private settings if they do not engage in mathematical activities regularly at work. Nienkemper and Grotlüschen (2019) examined the interactions between literacy practices, numeracy practices, and computer usage, and showed that ubiquitously writing adults also calculate ubiquitously, in both work and home contexts.

A comparison of numeracy practices, using data from comparative skill surveys from the mid-1990s to recent PIAAC data, shows a *decline*, i.e., lower levels of numeracy practices, over the last 20 years (Redmer and Dannath 2020), suggesting changes in the demands of different occupational clusters for numeracy skills. Overall, studies regarding numeracy practices (both in the social context and in terms of skill use) provide new insights into multiple types of factors that may contribute to growth, retention, or decline of skills throughout the lifespan. Such studies suggest that the types of tasks one has to engage with in everyday and work life can lead to personal growth but can also contribute to vulnerabilities in terms of skill formation, skills mismatch, or skills loss, and may create new learning needs in the area of numeracy.

### Evolving conceptualization of numeracy as functional and critical

Since the introduction of the term *numeracy* in the Crowther and Cockcroft Reports in the United Kingdom (Cockcroft 1982), many scholars and task forces have elaborated on and investigated what numeracy means, and have discussed the linkages between the world of numeracy and the world of (school-based) mathematics (e.g., Benn 1997; Coben et al., 2003; Condelli, et al. 2006; Gal et al. 2009; Geiger 2015; Ginsburg et al. 2006; Tout and Gal 2015; Tout and Schmitt 2002). Such reviews examined the many building blocks of numeracy, including cognitive elements (e.g., knowledge of content areas, skills) and meta-cognitive and dispositional aspects such as beliefs, attitudes, habits of minds, and related aspects.

One underpinning connection that has been highlighted in sources such as those listed above and many others (e.g., Geiger et al. 2015; OECD 2019a) is between numeracy and mathematics as a domain of knowledge. To be considered numerate, adults and young people are expected to know some (or a lot of) mathematics (and statistics), and be able to apply that mathematics within a real-world context.

The mathematical (and statistical) content usually encompassed within curriculum statements and guides that are of relevance to both school systems and adult learners of numeracy, usually covers four content areas (Kilpatrick 2001; Steen 1999): quantity and number; space and shape; relationship and change; and data and chance—or similar labels referring to key big ideas in mathematics and statistics. Similar labels are also used to describe the key content areas in PISA and PIAAC (Tout and Gal 2015) and in other assessments at national and international levels that pertain either to numeracy or to mathematical literacy.

In addition, two separate issues need to be highlighted as part of the conceptualization of numeracy. First, the earlier arguments presented in Sect. 2.4 imply that *practices* should become part of the conceptualization of numeracy, since they reflect how people's numeracy is enacted in the world and how it is continuously shaped by ongoing experiences. Furthermore, the conceptualization of numeracy should address both its *functional* and *critical* roles and its components. Both of these issues have ramifications for understanding how numeracy can be developed (in formal and informal contexts) and for understanding sources of vulnerability for adults and for numeracy learners in the twenty-first century, as explained below and in later sections.

It seems obvious to view numeracy from a functional perspective, for instance, to discuss it in terms of the application of arithmetical calculations or the understanding of simple mathematical ideas from the four content areas listed earlier (e.g., proportions, percentages, symmetry, area, volume, distance, and averages) and show their usefulness in the solution of everyday mathematical problems. However, the literature reiterates that as with any competency, numeracy encompasses a set of diverse skills, knowledge-bases, and other building blocks that range from simple to very advanced. Numeracy should, therefore, not be seen as pertaining to simple or basic levels of mathematical knowledge, but as a complex competency. The understanding of numeracy in adult education and as one of the factors that affect citizens’ well-being, increasingly encompasses the need for adults to be able to access, use, and apply a wide range of *advanced* mathematical skills and knowledge, including in a *critical* sense.

The notion of criticality is now routinely highlighted in virtually all frameworks that explore and define twenty-first century skills (e.g., Griffin and Care 2015), but has long-standing and diversified roots. An emphasis on critical reflection or interpretation is consistent with long-standing views in the area of adult numeracy, such as the concept of *critical numeracy* (Steen 1999), which connects with the interest in *critical mathematics* (Frankenstein 1989). For example, Johnston (1994) argued that:

To be numerate is more than being able to manipulate numbers, or even being able to ‘succeed’ in school or university mathematics. Numeracy is a critical awareness which builds bridges between mathematics and the real-world, with all its diversity (p. 34).

Early approaches argued that reading, writing, and calculation are not neutral and should be used to counter oppression. For instance, when curricula require students to learn how to invest money in order to generate high rates of interest, this teaches capitalism and not critical awareness (Freire 1996). Other notable perspectives that emphasize critical aspects of numeracy in order to promote socially, culturally, or economically responsible behavior, stem from international work in the area of ethnomathematics, such as that by D'Ambrosio, Rosa, and other scholars (Rosa et al. 2016).

Critical approaches to numeracy thus do not think only of the individual, but also focus on societal circumstances. Geiger et al. (2015, p. 535) have already argued that "an important aspect of becoming numerate is developing the capability to take a more critical view of the world—from personal, social, and political perspectives". Adults need to be able to interrogate and form opinions about trends in crime, unemployment, pollution, medical or environmental risks, and related issues, all of which require a critical understanding of civic statistics (Gal 2002; ProCivicStat Partners 2018).

Critical numeracy can be used to make claims for social justice. In this issue of *ZDM Mathematics Education*, this is illustrated as part of examining the *societal*/*community* role of numeracy for refugees and migrants (Jurdak 2020) or in Dìez-Palomar's (2019) study on women’s critical thinking about numeracy. Criticality is also essential in health and medical contexts, and in financial matters such as understanding and reacting to changes in social benefits, taxation or vital household expenses like power supply (Murphy 2019), and the monitoring of numerate environments (Evans et al. 2017). Gendered power relations in money management is another financial numeracy issue where a critical lens may be useful (Lave 1988; Redmer and Grotlüschen 2019).

Hence, to be considered numerate in the twenty-first century, adults and young people are expected both to know diverse aspects of mathematics, encompassing a broad and advanced range of knowledge, skills, and dispositions, and to be able to apply their knowledge within diverse contexts. The latest definition of numeracy for the planned 2nd cycle of PIAAC also highlights the importance of *reasoning critically* (Tout et al. forthcoming):

*Numeracy* is accessing, using and reasoning *critically* with *mathematical content*, information and ideas represented in multiple ways in order to engage in and manage the mathematical demands of a range of situations in adult life.

Overall, we argue that functional numeracy is *not* separate from critical numeracy—it is not a dichotomy, nor are they in competition with each other or should be seen as a hierarchy of skills. A functional perspective and a critical perspective are both relevant to each of the numeracy domains discussed earlier in Sect. 2.2. For many vulnerable adults, functional numeracy is vital and critical. Functional numeracy also is a stepping-off point for becoming critical (e.g., citizens who live under the poverty line or depend on government assistance for welfare, health, or food, need to monitor their income as well as their rights; see Smythe 2018).

On the one hand, it seems impossible to move towards critical awareness and critical reflection without having the functional skills necessary for functioning in a given society. On the other hand, people who lack certain functional skills do not automatically lack critical awareness. The two are *interdependent*. It is possible and necessary to be critical even without fully understanding the mathematical part first. For example, in an increasingly technologized age, algorithmic processes *of machine learning* are black boxes, even for those who produce them (Michael and Lupton 2016). For this reason, there is an emerging need to address both functional and critical aspects of numeracy as part of numeracy and mathematics education. Lack of attention by educators to either one of these issues can contribute to vulnerability in adult life.

## Numeracy within adult learning and education

How and where does people's numeracy develop, after they leave formal schooling? This section reviews selected issues and challenges associated with the learning of numeracy in diverse contexts, from a lifelong perspective. The section points to several sources of vulnerability, including at the system level and the individual level.

### About adult learning and education

*Adult learning and education* (ALE) is an umbrella term encompassing a wide range of contexts in which learning by adults may take place around the globe. Diverse classifications of ALE exist, using multiple lenses related to the organization, governance, or funding sources, how intentional the learning is, or what the purposes or motivations underlying the learning are, e.g., functional goals such as career promotion or work, leisure or well-being needs, or improving active citizenship and participation in community action (Jarvis 2004; Lee and Desjardins 2019).

A long-standing classification of ALE suggests that adult learning may occur in three contexts: formal, non-formal, and informal (Cedefop-European Centre for the Development of Vocational Training 2014); these are the contexts in which numeracy may develop as well. Although definitions may differ, we summarize them as follows:

- *Formal* learning involves learning that is intentional from the learner's perspective, occurs in an organized context created by an institutional actor, and usually leads to a formal credential, diploma, or certificate. Examples include a vocational education course leading to a training qualification, or an adult education program operated by a local educational body that offers courses, possibly including a mathematics course, leading to a high-school equivalency diploma.
- *Non-formal* learning is embedded in planned activities that are not explicitly designated as formal education and do not usually involve credentialing, yet still contain a substantial learning element, which is intentional from the learner's point of view. Non-formal learning contexts offer many opportunities for lifelong numeracy learning, as when taking part in counseling to over-indebted persons (Angermeier and Ansen 2020), attending a company workshop or a retirement planning lecture at a community center, or participating in programs for economic empowerment of women or minority groups.
- *Informal* learning may occur spontaneously, i.e., learning is usually non-intentional or governed by a structured educational process, but occurs as part of daily activities and is experiential in nature. Indeed, a well-known line of research refers to informal learning of mathematics, as in the literature on everyday cognition or 'street math' (Lave 1988). Several papers in this issue of *ZDM Mathematics Education* discuss related issues regarding numeracy practices, all of which offer opportunities for informal learning.

A newer typology of education and training provision (Boeren and Whittaker 2018) sheds more light on types of adult learners in formal education as it integrates some older typologies and new lifelong learning policy initiatives in Europe and elsewhere. This typology consists of seven categories: (1) Basic skills and basic education, (2) 'Second chance' education at upper secondary level, (3) Post-secondary vocational-technical education, (4) Apprenticeships, (5) Training that forms part of active labor market policies, (6) Workplace or job-related learning, and (7) Personal or social learning.

Numeracy learning may occur in all categories listed by Boeren and Whittaker that intersect with formal, non-formal, and informal contexts. Many of these categories or contexts are examined by papers in this issue of *ZDM Mathematics Education*, and some by papers in a prior 2015 *ZDM* issue on numeracy in general.

In reflecting on sources of vulnerability for adult numeracy learners, it is important to look at adult (numeracy) education through a *systemic* lens, as there may be gaps between supply and demand of learning opportunities, especially formal and sometimes non-formal ones. Adult education services (which involve curricula, classrooms, teachers and teacher preparation, and credentialing mechanisms) are provided via formal organizational structures and are grounded in public policy or organizational policy. Policy and its actual enactment, whether at the national, local, or program level, is where a discussion of vulnerabilities in numeracy education is most pertinent. After all, policies are modifiable, and depend in large parts on shifting political and social preferences or on economic possibilities and pressures.

Indeed, as Desjardins et al. (2016) argue, adult education and learning policies (and funding levels and mechanisms) vary widely across countries. The recent Global Report on Adult Education and Learning (GRALE; UNESCO Institute for Lifelong Learning 2019, p. 15) showed that *participation rates* in adult education also vary widely across countries, and argued that chances to participate are considered “shockingly unequal”. This points to systemic sources of vulnerability in terms of the *opportunity to learn* and raises new questions about the factors underlying such patterns.

### Numeracy and numeracy teachers in adult education

The above typologies, and the various international perspectives on the future of education and training for adults (e.g., European Qualifications Framework for Lifelong Learning; OECD Skills Strategy [OECD 2019b), suggest that the organization and delivery of formal and non-formal education and learning opportunities for adults is very diverse, more so than the provision of mathematics education within compulsory or tertiary education systems.

To illustrate the multifaceted nature of the organization of formal numeracy-related adult education and learning, and teachers' qualifications in this regard, this subsection describes the situation in several countries. We start with Germany, which has the largest population among European Union countries, and according to the latest GRALE (UNESCO Institute for Lifelong Learning 2019) has one of the most developed systems of adult education, hence it is discussed in more detail below.

In Germany, adult education nowadays is formalized in the constitutions of its 16 federal states, not on the overall federal level. Most federal states have laws that allow for financial support of literacy and basic adult education for vulnerable subpopulations. Furthermore, Germany is currently implementing a ‘decade for literacy and adult basic education’ policy (2016–2026). The relevant policy claims that literacy is the ability to read, write, and compute (i.e., following a traditional view held by UNESCO, subsuming numeracy within literacy) but its literacy domains include financial, digital, health, and civic noted earlier. Nevertheless, only one out of 25 projects funded by the literacy decade initiative focuses directly on adult numeracy, covering research and training on financial literacy (Mania and Tröster 2015).

Unlike other countries, adult basic education in Germany is governed and funded separately from other types of adult learning described above by Boeren and Whittaker (2018). It includes second-chance schools and vocational education (funded by education ministries), remedial education for adults (funded by the pension system), language provision (funded by the interior ministry) and training in unemployment agencies (funded by the labor ministry). This implies the possibility of fragmentation of curricular schemes and heterogeneity of teacher qualifications. For example, trainers working in unemployment agencies do not need a teaching certificate, even though courses for job seekers have components on quality control and may involve some workplace mathematics, whereas trainers in some other adult education services (e.g., the German folk high schools) do not need to have formal certification, but can acquire qualifications through *non-formal* schemes.

Many other countries with established adult education systems show a similar fragmentation of adult education learning schemes and diversity in the demands for teacher qualification in mathematics and numeracy. For instance:

- In the USA, a country with close to four million learners in federally-funded adult education programs, an early national survey (Gal and Schuh 1994) found that 85% of adult numeracy instructors were part-time; fewer than 5% of teachers in programs providing numeracy education were certified to teach mathematics, and few had received any pre-service training in mathematics instruction. Later on, Condelli et al. (2006) confirmed this picture. Smith and Gillespie (2007) summarized the characteristics of adult basic education teachers in the USA as follows: they work mostly part-time, they leave the field more often than do school teachers, few have formal qualifications in teaching adults, though many are qualified and have taught in K-12, and they are not consistently funded to participate in in-service professional development.
- In the UK, a review of the professional development and qualifications of adult educators revealed the inconsistent and poor background of teachers and the lack of systematic professional development opportunities (Morton et al. 2006).
- Going beyond Germany, USA, and the UK, an OECD comparative review about the background, experience, and knowledge of adult literacy and numeracy teachers shows a consistent pattern across many different European countries (Windisch 2016): Few teaching staff have formal qualifications in adult literacy and numeracy pedagogy.

Based on such literature reviews and available data about numeracy (and literacy) teaching and programs in several high-income countries, it appears that numeracy is infrequently taught as a stand-alone subject, and more often appears under different names, i.e., as part of language provision, initial vocational education, further training for unemployed, and so forth. That said, second-chance schools, which provide basic qualifications to adults who have had little formal schooling (e.g., due to being migrants or refugees) and seek school completion certificates, often do teach mathematics as a separate subject, but following the standard curriculum for school students (Pavic and Černja 2019). Furthermore, going beyond formal education to non-formal numeracy education, little is known about the content provided in in-house training that companies or worker unions provide for their employees or members, nor about teacher qualifications in such contexts (Kelly 2019).

Overall, the realities described above point to many systemic factors that can create sources of vulnerability from the point of view of numeracy learners and education providers alike, e.g., in terms of teacher qualifications and training, curriculum cohesion and relevance to adult learners. These realities show the need for much further systematic research that will map numeracy education pathways and evaluate the relative quality of education services across the many contexts in which adults may learn numeracy or mathematics, both formal and non-formal.

### Participation, achievement, and dispositional or affect-related factors

The adult education literature has paid much attention to *barriers to participation* and to factors affecting *persistence, engagement,* and *dropout* (Comings 2008). Many factors have been discussed in this regard: *person- or learner-related* (e.g., cognitive preparedness, psychosocial characteristics, learning habits, motivation), *technical* (e.g., schedules, location, transportation), *program-related* (e.g., quality of curriculum, availability and quality of teachers), and *policy-related* (Lee and Desjardins 2019). These factors and issues are of interest to any educator, and are raised because of their obvious connection to the intersection between numeracy, adult education, and vulnerability.

One key learner-related issue that numeracy educators often encounter is linked to the realization that adult learners may bring to their numeracy studies specific dispositional or affective factors, which may have both negative and positive aspects (Safford-Ramus et al. 2016). On the negative side, adults may bring negative memories, mathematics anxiety or phobia, low self-efficacy, unproductive beliefs (e.g., about mathematics and its usefulness), and other negative attitudes, feelings, and self-perceptions. These and similar factors are often the result of prior experiences with mathematics instruction and encounters with mathematics teachers at the school level (Hannula et al. 2019), but can also result from negative encounters or 'unsuccessful' numeracy practices outside of formal schooling.

Such attitudes and beliefs stand in contrast to the sense of 'at-homeness with numbers' that is desired of school graduates (Cockcroft 1982) and can interfere with one’s motivation to develop new mathematical skills or to tackle mathematics-related tasks as an adult (Gal et al. 2009). Overall, one's experiences in formal schooling may affect the motivations and identity one develops which, in turn, may affect one's behaviors (or avoidance) and practices as a learner and user of mathematical knowledge. For example, mathematics anxiety, which has been well researched for decades, has been shown to have a significant impact on performance in mathematics (e.g., Ma 1999).

Of course, dispositional or affect-related factors must not be seen only from their potential negative side (which is understandable in the light of this paper's focus on vulnerability), but through a positive lens as well. Positive dispositions and motivations, or high-self efficacy, are key factors that can contribute to engagement and high achievement (Hannula et al. 2019). To illustrate this, Swain (2005) reported, based on a qualitative study of adult learners returning to study numeracy in college, that learners had multiple complex motivations, inextricably linked to the learners' identity. He concluded that mathematics studies do not have to be *functional* in order to capture students’ interest, involvement, or imagination.

In sum, dispositional and affect-related factors illustrate unique sources of vulnerability (or success) of adult numeracy learners, apart from literacy or knowledge-related factors discussed earlier. Such factors may affect adults' willingness to arrive at the classroom door, persistence, and actual achievement. In addition, dispositional factors may impact engagement with (or avoidance of) numeracy tasks and resulting numeracy practices in the real world, beyond the classroom walls. The research literature on this issue is, however, patchy at best with regard to adult learners, especially in terms of longitudinal or mixed-method designs that track the development of dispositions and affect over time, or analyses of effective interventions that can undo and overcome negative dispositions or beliefs related to mathematics and its learning, which learners bring from their school days.

## Vulnerability and vulnerable groups

### About vulnerability

Vulnerability is a multi-faceted term. In feminist discourse, vulnerability is regarded as an *essential* human condition, i.e., it is understood as a 'conditio humana' that states that every human being depends on relations with others and is thus vulnerable on all levels—in interaction with others, with institutions, and on a societal level (Butler 2016). Yet, the essence of vulnerability is also that it has an *empirical* aspect, i.e., it refers to the possibility and probability of getting hurt, where some members of a society are better protected than others from actually getting hurt, as revealed by empirical data (e.g., official statistics or comparative studies). Thus, vulnerability captures a seeming paradox that relates to the status of social groups that are part of society but are at increased *risk* of social, political, or economic exclusion or disadvantage (Streich 2009), though are *not necessarily* actually being excluded or disadvantaged (or are on a continuum of severity).

As the World Health Organization and social sciences originally introduced the concept/term, vulnerability relates to subpopulations that are systematically exposed to factors (e.g., lack of clean water, unsafe living or working conditions, poor nutrition, very low income) that increase their risk to become sick, reduce life expectancy, slow physical development or growth, etc. Streich (2009) transferred the term vulnerability from health research to social work and social services. His concerns refer, for instance, to the dangers of being overly indebted, exclusion from banking, difficulties with housing because of low or no income, as well as vulnerability to unemployment or unstable work due to poor qualifications and skills or being part of a minority group.

Adults with lower numeracy skills are often described as vulnerable, marginalized or at high risk of being excluded from labor markets and social life (OECD 2019b). Data shows, however, that the majority of low numerate or low literate adults are employed and integrated into family and social life (Grotlüschen et al. 2016), at least on some level, i.e., they are not necessarily excluded from labor markets and social life, though they are at higher risk in this regard (Jonas 2018; OECD 2019a). This paradox is difficult to capture with a dichotomous view of persons as being either vulnerable or not, or being either in an inclusion or exclusion binary status. Moreover, this dichotomy fosters stereotyping and stigmatization, deficit orientation and ‘blaming the victim’, problems that have long been discussed in literacy research. Furthermore, vulnerable subpopulations account for a sizeable percentage of the overall population, not a negligible minority. The clearest example is women, who are still in many ways excluded from social power and income sources, but constitute half of the world population.

It thus seems appropriate to move on to a theoretical notion of vulnerability as a continuum, which is applicable for different aspects of life. For example, one can be included and function well in one area (e.g. family life) yet be vulnerable in other areas (e.g. housing). Vulnerability should therefore not be seen as a stigmatizing term, but be defined as a continuum that extends from comfortable interrelatedness (given the feminist idea of vulnerability as a normal human condition) through more precarious social positions or increasing risk of exclusion. The other end of the continuum would be the factual exclusion, e.g., long-term unemployment, which gradually leads to complete dropping out of the formal labor market. Educational needs and policy-based interventions should then be considered accordingly, aiming among other things to empower diverse vulnerable groups (Benn 1997; UNESCO 2016).

### Rationale—why vulnerability and adult numeracy are coupled

Overall, research shows that skills such as literacy and numeracy lead to desirable social and economic outcomes, for example in the area of income and job security, as well as so-called wider benefits of learning, such as health and socio-political participation (OECD 2019a). This general and recurrent finding is noted and confirmed by many studies (Hanushek and Woessmann 2007; Liu et al. 2019). Educational services and training programs often target marginalized populations via national skills strategies (OECD 2019b) and various types of adult education programs. Numeracy is a foundational element in all such programs, alongside literacy and language skills.

The discussion of vulnerability also raises moral questions since it calls on policy makers and social institutions to reduce the vulnerability of certain groups. For example, they have an ethical responsibility to combat poor living conditions or poor nutrition and improve health outcomes, or help those with needs due to skill shortages and employment gaps. The improvement of numeracy in specific domains of life discussed in Sect. 3, (i.e., health numeracy, financial numeracy, digital numeracy, civic numeracy, and workplace numeracy), are essential ingredients in this regard. These domains are, therefore, further revisited in the next subsection, and expanded beyond their initial description in Sect. 2.2.

### Vulnerability as captured in adult numeracy research

Vulnerability is mostly captured as a single characteristic of a group, but some cases involve an intersection, as with migrant women or unemployed youth. Numeracy stands at the center of the discussion, as it may affect the vulnerability and possibility of reducing the vulnerability, of groups studied in all papers in this issue of *ZDM Mathematics Education*, including:

- Low-numerate adults and students (Fonseca; Heilmann; Liu; Prince and Frith; Redmer and Dannath)
- People in financial debt (Angermeier and Ansen)
- Gender, age and elderly (Dìez-Palomar; Civil et al.; Zeuner et al.)
- Migrants and refugees (Jurdak; Nortvedt and Wiese; Civil et al.; Lüssenhop and Kaiser)
- Aboriginal or indigenous persons (Miller et al.; Jorgensen)
- People with learning differences/difficulties (Schreiber-Barsch et al.)
- Imprisoned persons (Reder)

The remainder of this section examines three questions, framed against the results and theoretical arguments stemming from the papers in this issue of *ZDM Mathematics Education*, and building on earlier sections of this paper. First, we examine whether the findings support the need to address separate numeracy domains, discussed in Sect. 2.2, in ways that also link with sources of vulnerability. Next, we examine whether the reported studies support ideas discussed in Sect. 2.3 regarding linkages between numeracy and literacy. Finally, we show how the findings support the proposal presented in Sect. 2.5 regarding the need to address both functional and critical aspects of numeracy of adults, and illustrate aspects of numeracy practices, a notion discussed in Sect. 2.4, and some aspects of adult learning and adult (numeracy) education, raised in Sect. 3.

#### How is vulnerability related to separate numeracy domains?

This subsection illustrates how key numeracy domains described in Sect. 2.2, i.e., health numeracy, financial numeracy, digital numeracy, and civic numeracy, are reflected in papers published in this issue of *ZDM Mathematics Education,* in ways that also point to related vulnerabilities and to the need for both functional but also critical numeracy skills. This analysis has implications for conceptualizing targets for adult numeracy education. (Regarding workplace numeracy, see Hoyles et al. 2010; Straesser 2015).

Several new findings confirm the relevance of *health numeracy* in adult lives. This term represents the degree to which individuals have the ability to access, process, interpret, communicate, and act on numerical, quantitative, graphical, bio-statistical, and probability-based health information (Golbeck et al. 2005). Descriptions from qualitative data (Zeuner et al. 2020) on elderly adults indicate that health-oriented activities demand numeracy practices, e.g., probability checks (chances of recovery and risks of an operation), scaled self-observations (pain protocols), dosage tasks, or expenses and bonuses claimed from health insurers. Large-scale data confirm the relevance of health numeracy to adults' well-being. Based on a longitudinal study (PIAAC-L), Heilmann (2020) showed a relationship between aspects of health-related behavior (non-smoking, exercise, health-conscious nutrition) and basic numerical practices such as estimating measurements and calculating simple averages, as well as more complex numerical practices such as the interpretation of statistical data.

Regarding the relationship between financial literacy and *financial numeracy*, substantial research shows that numeracy is even more relevant than language proficiency (Evans et al. 2017; Lave 1988; Redmer and Grotlüschen 2019). For example, debt counselors who work with over-indebted people seeking advice (Angermeier 2019; Angermeier and Ansen 2020) point out that commercial and service providers often present product descriptions (e.g., credit offers, installment purchases, telephone and electricity bills) that make price comparisons difficult and require advanced numeracy skills. Grotlüschen et al. (2019b) show that complex financial decisions (retirement plans, tax returns) are less likely to be made by low-literate adults, while numeracy practices of over-indebted people (Grotlüschen et al. 2019a) involve the intense use of finance-oriented numeracy practices and skills. Research also shows that many people use online banking in a functional manner, but few feel convinced that they can actually judge the risks and chances properly (Buddeberg 2020).

*Digital numeracy* is not about using technology in instruction, although this is of course important in and of itself (Geiger 2015). Rather, digital numeracy refers to skills and dispositions required in today's’ quantitative, data-rich world (Evans et al. 2019). New challenges range from understanding ubiquitous data collection, algorithmic decision making, and uses of 'big data' in prediction systems (Michael and Lupton 2016). Citizens need to understand the potential contribution of such developments to inequality or discriminatory use, e.g., health insurance based on the probability of an improved situation (Smythe 2018), and to distinguish advertisements from information and understand disinformation (European Commission 2018). Zuboff’s (2019) recent theory on surveillance capitalism calls for critical awareness of how human behavior is predicted and modified based on personal data to benefit leading internet companies. The upshot is that citizens who do not develop digital numeracy and a critical stance in this regard are vulnerable, e.g., to actions by various service providers.

*Civic numeracy* covers multiple issues, starting with the understanding of statistical data (Evans et al. 2019) and civic statistics (ProCivicStat Partners 2018) in order to monitor and engage with societal issues (e.g., rising crime or pollution). Civic numeracy also covers the contribution of numeracy to social justice advocacy (Jurdak 2020) and understanding of power relations (Yasukawa et al. 2018). Quantitative knowledge is relevant when claiming a fair share and fair treatment (Kalman and Solares 2018) and for adult empowerment (Dìez-Palomar 2019). Schreiber-Barsch et al. (2020) argue that elderly people and people with disabilities are under-represented in large-scale assessments, and their practices only gain visibility and social recognition when they also appear in measurements. The upshot is that citizens who lack both functional and critical aspects of civic numeracy may be vulnerable or open to inequality or lack of social power in various spheres of life.

#### Do numeracy, literacy, and language interact?

Linkages between numeracy/mathematics and literacy/language were already noted in Sect. 2.3. Findings presented in the papers in this issue of *ZDM Mathematics Education* support and speak to the need to address such linkages. Language matters for both numeracy skills (Prince and Frith 2020) and practices (Civil et al. 2020; Fonseca 2020; Jorgensen 2020).

These findings show that numeracy is dependent on literacy and language skills, although it may be theoretically assumed that literacy and language practices also contain aspects of numeracy, and mesh in the many contexts involving 'document literacy' (Kirsch et al. 1998). For instance, newspaper and internet readers come across numbers and mathematical words in texts, graphs, tables, and charts and encounter information and arguments using proportions, chance, amounts, and trends. Functional literacy practices like filling in forms, searching for information online, and writing formal letters regularly require a wide range of numeracy practices. These theoretical arguments can benefit from additional empirical support.

#### How are functional and critical aspects of numeracy represented?

Findings presented in several papers in this *ZDM Mathematics Education* issue support the argument raised in Sect. 2.5, according to which functional and critical aspects are both essential for adults, and are intertwined. Critical aspects focus on both numeracy and literacy as societal capabilities (Zeuner 2013) that individuals and groups can use to examine and challenge their positions in a society and the overall distribution of goods and power (Freire 1996). Several numeracy researchers focused their approach on this critical aspect (Dìez-Palomar 2019; Geiger et al. 2015), others include motivational aspects (Liu 2020). Some papers in this issue, however, discuss numeracy skills and practices also from a functional perspective, e.g., for incarcerated adults with a restricted numerate environment (Reder 2020), indigenous learners who benefit from educational vocational programs (Jorgensen 2020; Miller et al. 2019), or refugees in schooling preparation classes or other educational settings (Lüssenhop and Kaiser 2020; Nortvedt and Wiese 2020).

## Discussion and implications

The need for further attention to the numeracy of adults and young people and to its development around the globe, with a focus on people deemed vulnerable, seems essential and timely in light of an increasing global interest in numeracy as part of the Sustainable Development Goals and by multiple policy-driven international assessment programs (PIAAC, PISA and others). Accordingly, this paper provided a critical examination of multiple aspects of the conceptualization of numeracy and its changing roles and building blocks, discussed five specific numeracy domains, reflected on issues and gaps within adult education systems that may affect numeracy learners, and problematized the notion of vulnerable learners of numeracy.

Some key insights from the reviews of the above topics can be summarized as follows:

- Numeracy is as ubiquitous as literacy, and as multifaceted. It is time to differentiate between numeracy domains and consider effective teaching methods for each.
- Literacy and language, and numeracy and mathematics, are interrelated at a level that requires renewed and deeper consideration by researchers as well as by numeracy and mathematics teachers.
- Functional and critical approaches to numeracy are both intertwined and essential in all five numeracy domains reviewed earlier, with no hierarchy and no dichotomy between functional and critical aspects.
- The view of vulnerability regarding adult numeracy and numeracy education should be broadened to cover multiple subpopulations and encompass both personal, societal, and systemic sources of vulnerability.

Going beyond these insights, the findings we reviewed and those reported in other papers in this issue of *ZDM Mathematics Education*, lead to several conclusions, as follows. First, to be considered capable or competent in any aspect of life, and in particular concerning situations with any mathematical or statistical aspect, whether explicit or implicit, requires the integration of multiple personal systems. Cognitive skills and knowledge bases are core elements of numeracy from the point of view of educators and curriculum developers, and lead the conceptualization, teaching, and assessment of numeracy. Acting in a numerate way, however, also involves meta-cognitive elements (e.g., reflectiveness) as well as non-cognitive or semi-cognitive aspects (e.g., emotions, motivations, value judgments, personal views).

Second, viewing numeracy as a competence has additional important implications, because a competence is a *dynamic* structure (Rychen and Salganik 2003) that depends on individual opportunities to practice a behavior (i.e., external affordances), as well as on actual behaviors and their results (i.e., feedback and internal change via reflection). Furthermore, as this paper and other papers in this issue demonstrate, numeracy practices occur in social and cultural contexts, and are affected by individuals' personal histories and prior learning trajectories. Numeracy and numerate behavior have also been shown to be interdependent both on language skills and on literacy practices. For these reasons, adult numeracy is a challenging area as a target for educational action and research.

Third, numeracy should have a similar standing to that of literacy, due to humanistic, theoretical, civic, and functional considerations. This means, however, that numeracy must also be differentiated in the formulation of (adult) education policies and practices, as well as in educational research. This paper pointed to five domains in which separate but connected numeracies are needed by adults, related to digital, financial, health, civic, and the broad area of workplace numeracy. Each of these domains requires somewhat different knowledge bases, dispositions, and practices, although some issues cut across multiple domains. These five domains may be seen as *functional*, calling for pragmatic responses, yet they all also involve *critical* elements since they require the ability (and inclination) to raise questions, reflect critically, and communicate effectively about thoughts and concerns. Furthermore, based on Gal (1997), each of these five domains requires not only *computational*, but also *interpretive* and *decisional* reactions, hence present higher task demands than may seem from a functional view of numeracy, and may require different teaching/learning processes.

Fourth, given the complex terrain involving multiple *numeracies* and *numeracy practices* of diverse types of adults, this paper discussed a broad range of vulnerabilities that adults face in functional and societal contexts, as well as when they are learners (especially in formal education). Some of the vulnerabilities are well known in the literature and pertain to personal characteristics (e.g., minority status, learning difficulties) or to situational and social barriers (e.g., economic conditions, social exclusion). Sections 3 and 4, however, contributed additional sources of vulnerability related to negative affective or motivational baggage that adults bring with them when they enter a learning program or engage with various numeracy tasks, as well as poor literacy skills. Other barriers are *systemic* and may pertain to the characteristics or resources of the learning programs that adults may enter. They also pertain to the qualifications and practices of teachers, their understanding of the unique aspects of teaching adults (rather than school children), and the understanding of how to promote numeracy–literacy links. All of these vulnerabilities can inform goals and policies in adult numeracy education.

Fifth, as the twenty-first century progresses, as discussed in Sect. 2, the nature of the numeracy situations in the adult world is changing across the globe. Rapid technological, scientific, economic, and social changes and developments increasingly require adults to have more critical and reflective reasoning skills and the ability to recognize, interpret, and understand a broadening spectrum of manifestations or contributions of mathematics, statistics, and numeracy across a wide range of life domains (Griffin and Care 2014). This is reflected, for instance, in the barrage of communications and data presented to the public worldwide regarding probabilities, risks, and quantitative projections associated with the recent Corona-virus (COVID-19) pandemic, and the resulting need for many interpretive and decisional reactions from citizens from all walks of life. Educators who will not attend to the critical side of numeracy may contribute to their learners' vulnerability.

Finally, promoting adult numeracy skills and developing effective learning systems requires attention to the fact that the majority of adults in any country are *outside* the reach of formal education systems. Thus, planning of learning opportunities should take into account both formal, non-formal, and informal learning pathways. The distinction between these learning pathways is in fact becoming blurred, given emerging technologies and the rise of open digital resources (e.g., MOOCs) and instructional videos on social platforms and websites of public and private actors, which enable self-governed informal learning, without a set curriculum. Hence, it is necessary to broaden the conceptualization of what may be considered adult education in numeracy.

### Research realities

The conclusions sketched above open up a variety of research questions and knowledge needs; while some may appear general, most have elements that are unique to the area of numeracy. It is, however, quite common in many countries that the focus in research related to adult education and training is on *literacy*. Numeracy is often forgotten, as review papers published over the last two decades have illustrated.

In a 2002 analysis, Tout and Schmitt found that while there was a high level of research into both mathematics education (in schools) and into adult basic education in the US, less than 1% of any such reported research addressed numeracy or mathematics within adult basic education. Tout and Schmitt (2002) therefore called for further research about how adults think mathematically, what resources they bring to bear in approaching and solving problems, and what instructional interventions may best support the development of numerate thinking.

Similar conclusions were reached by Condelli et al. (2006) in a federally-funded systemic review of research on adult numeracy education in the USA. Their recommendations for research included the following five areas:

- Evaluate instructional frameworks and theories of adult mathematics learning.
- Identify and evaluate specific instructional practices.
- Study how adults learn mathematics in class.
- Explore the role of learner attitudes, affect, and experience (i.e., practices).
- Examine learners and students with learning disabilities or who study in a language other than their home language.

A decade later, an analysis sponsored by OECD (Windisch 2016) concluded that few intervention studies across OECD countries have been conducted and little is known about strategies that are most effective in improving adult foundation skills. This study argued that tackling serious literacy and numeracy weaknesses is challenging because low-skilled adults are diverse and require different, well-targeted interventions.

### Research directions on adults and numeracy

Given the research realities and gaps listed above, we see many possible directions for future research, given the need to inform policy regarding 'what works' (or 'could work') in adult numeracy programs, as well as to inform planning of educational interventions at the program or teacher level. Examples of research-worthy questions include, but are not limited to the following broad topics:

1. What is the impact of dispositional and affective factors (broadly viewed, including attitudes, purposes and motivations for learning, beliefs, identity, etc), which may have both negative and positive aspects, on participation, retention, persistence, and engagement by learners of numeracy?

2. What are the numeracy practices (both productive and unproductive) of vulnerable groups (several subtypes were described in this paper) and how should such practices be considered when planning and implementing instruction?

3. What teaching (and assessment) practices can promote the teaching and learning of critical aspects of numeracy, and what is the role of new technologies in this regard?

4. What content knowledge (CK) and pedagogical content knowledge (PCK) are required by teachers in the area of adult numeracy, and what professional development schemes can promote such knowledge?

5. What curricula or teaching practices can enhance literacy-numeracy linkages, i.e., enable learners to effectively engage with the literacy aspects of numeracy?

6. Since learning of numeracy and numeracy practices occur in many *non-formal* as well as *informal* life contexts (e.g., health, financial, digital, civic, workplace), to what extent are the skills and dispositions that adults bring from such contexts recognized and capitalized on by teachers, curricula, and teaching practices in formal numeracy education programs?

Note that the six questions listed above are sampled from broader clusters of possible questions: The first two illustrate research directions related to *learners*, and the next three illustrate questions related to *teachers, programs, and teaching/learning processes.* The last question integrates all of the above, but points to the need to shed more light on *non-formal* and *informal* learning contexts, which abound but to date are mostly poorly researched in terms of their connection with numeracy teaching/learning in formal education.

Such and related research directions may gradually contribute to theory-building in the area of adult numeracy, and also inform the practice of numeracy education for both adults and younger people. Research on such issues seems urgent, given demographic shifts and migration (UNESCO 2018) and other social and economic trends (OECD 2019b), rapid technological changes, and the international emphasis on creating *sustainable* changes in education and skills (UNESCO 2016). All these may necessitate an expansion in the thinking on how to address 'vulnerability' as part of numeracy education, both in high-income as well as in all of the many other world countries, given that, as argued in this paper, there are many sources to vulnerability.

Overall, the arguments and findings discussed in this paper have been selective, due to the breadth of the areas involved. Yet, taken together, they demonstrate that numeracy knowledge, skills, and practices (and related dispositions), in parallel with literacy skills and practices, should be one of the key targets for policy-driven educational interventions that aim to improve the social and economic status, and overall well-being of low-skilled or otherwise vulnerable adults. This paper highlights the fact that the roles and purposes of adult numeracy are complex, yet are becoming more multifaceted and dynamic in a rapidly changing twenty-first century. Thus, the conceptualization of the numeracy skills and practices that adults need should be continuously monitored and adjusted by policy bodies, researchers, and practitioners involved in adult learning and skills, and no less so by those interested in mathematics and statistics education in schools, STEM, and vocational contexts.
